# Baroreceptor denervation reduces inflammatory status but worsens cardiovascular collapse during systemic inflammation

**DOI:** 10.1038/s41598-020-63949-x

**Published:** 2020-04-24

**Authors:** Mateus R. Amorim, Júnia L. de Deus, Camila A. Pereira, Luiz E. V. da Silva, Gabriela S. Borges, Nathanne S. Ferreira, Marcelo E. Batalhão, José Antunes-Rodrigues, Evelin C. Carnio, Rita C. Tostes, Luiz G. S. Branco

**Affiliations:** 1 Dental School of Ribeirão Preto, 14040-904, University of São Paulo, São Paulo, Brazil; 2 Ribeirão Preto Medical School, 14049-900, University of São Paulo, Ribeirão Preto São Paulo, Brazil; 3 Nursing School of Ribeirão Preto, 14040-902, University of São Paulo, Ribeirão Preto São Paulo, Brazil

**Keywords:** Immunology, Physiology, Diseases

## Abstract

Beyond the regulation of cardiovascular function, baroreceptor afferents play polymodal roles in health and disease. Sepsis is a life-threatening condition characterized by systemic inflammation (SI) and hemodynamic dysfunction. We hypothesized that baroreceptor denervation worsens lipopolysaccharide (LPS) induced-hemodynamic collapse and SI in conscious rats. We combined: (**a**) hemodynamic and thermoregulatory recordings after LPS administration at a septic-like non-lethal dose (**b**) analysis of the cardiovascular complexity, (**c**) evaluation of vascular function in mesenteric resistance vessels, and (**d**) measurements of inflammatory cytokines (plasma and spleen). LPS-induced drop in blood pressure was higher in sino-aortic denervated (SAD) rats. LPS-induced hemodynamic collapse was associated with SAD-dependent autonomic disbalance. LPS-induced vascular dysfunction was not affected by SAD. Surprisingly, SAD blunted LPS-induced surges of plasma and spleen cytokines. These data indicate that baroreceptor afferents are key to alleviate LPS-induced hemodynamic collapse, affecting the autonomic control of cardiovascular function, without affecting resistance blood vessels. Moreover, baroreflex modulation of the LPS-induced SI and hemodynamic collapse are not dependent of each other given that baroreceptor denervation worsened hypotension and reduced SI.

## Introduction

Sepsis is a highly prevalent disorder affecting 31.5 million people worldwide, with a mortality rate of 5.3 million deaths every year^1^. Considering the severity of sepsis and its pathophysiological complications, studies have focused on the demonstration of mechanisms of systemic inflammation (SI) in search of therapeutic strategies help manage signs and symptoms for the treatment of this critical condition^2–4^. Systemic administration of lipopolysaccharide (LPS) induces SI, and has been widely used to induce changes observed during sepsis, such as exacerbated production and release of inflammatory markers, catecholamines, hormones and nitric oxide (NO), associated with hypotension, tachycardia, and hypothermia followed by fever^5–9^. These hemodynamic and thermoregulatory responses to LPS are similar to those observed in human beings during sepsis^10^.

The classical role of baroreceptor afferents are related to the homeostatic control of blood pressure in health and disease^11–13^. Rats with surgically removed baroreceptor afferents [sino-aortic denervation (SAD)] displayed higher variability of the mean arterial pressure^11,14,15^, demonstrating the mandatory role for baroreceptor afferents integrity. More recently, it has been shown that baroreceptor afferents play a key role of mediating LPS-induced cardiovascular collapse^16–19^. Moreover, in SI, electrical baroreflex stimulation in rats has been reported to blunt LPS-induced production of cytokines in the hypothalamus^20^, indicating an anti-inflammatory role played by aortic depressor nerve stimulation. However, the involvement of the baroreceptors afferents in the systemic inflammatory status has not received the same attention, neither its putative link with the cardiovascular system during SI.

In this study we examined in an integrated and comprehensive matter the role of baroreceptor afferents integrity in LPS-induced classical inflammatory cytokines surges [in plasma and spleen – effector organ of the splenic anti-inflammatory reflex^2,21^], hypothermia and fever, as well as the relation of the inflammatory status with cardiovascular function in rats. We hypothesized that baroreceptor denervation worsens LPS-induced hemodynamic collapse (linked with autonomic and vascular dysfunction) and increases SI (assessed by cytokines surges in plasma and spleen) in rats.

## Results

### Cardiovascular and thermoregulatory changes during LPS-induced SI is dependent on sino-aortic afferents integrity

**Figure 1:**
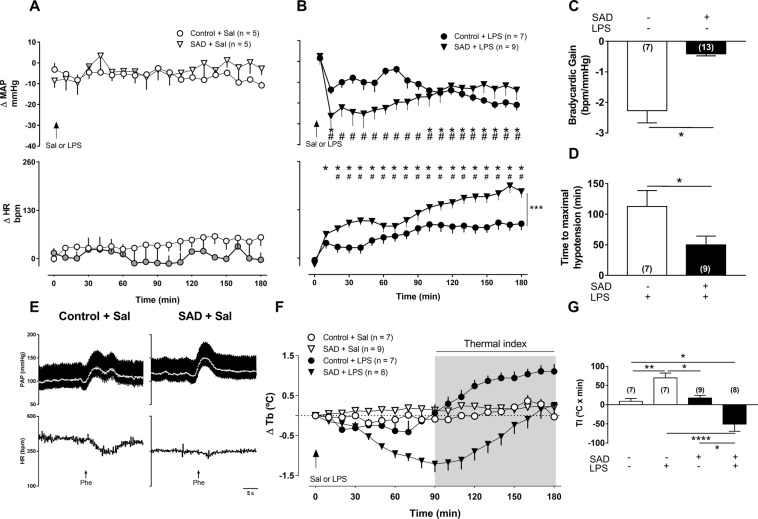
Effects of sino-aortic denervation (SAD) on mean arterial pressure (MAP), and heart rate (HR) of saline (Sal)-treated rats (panel A) and rats with SI (panel B, LPS, 1.5 mg.kg^−1^). Bradycardic gain during stimulation of the arterial baroreceptors (panel C) and maximal hypotensive response to LPS-induced SI (panel D). Representative recordings showing PAP, MAP (white line; top) and HR responses to i.v. injection of phenylephrine (Phe; panel E). Δ Tb (panel F) and thermal indexes (panel G). Results are presented as and mean ± SEM. *,^#^p < 0.05 compared with time zero and ***p < 0.0001 difference between groups using the two-way ANOVA with Tukey’s post hoc test (panel A and B). *p < 0.05 using the unpaired t test (panel C and D). **p < 0.01 using the one-way ANOVA with Tukey’s post hoc test (panel G). Control + Sal (*n* = 5–7), Control + LPS (*n* = 7–9), SAD + Sal (*n* = 5–9), and SAD + LPS (*n* = 8–9).

First, we investigated whether LPS-induced responses in mean arterial pressure (MAP) and heart rate (HR) were affected by the surgical removal of the arterial baroreceptors (SAD). MAP (P = 0.5189) and HR (P = 0.2197) were similar in Control + Sal and SAD + Sal animals throughout 180 min (Fig. 1A) after LPS or saline administration. LPS-induced fall in MAP was significantly higher (P < 0.0001) and occurred earlier (P = 0.0413) in SAD + LPS in comparison to Control + LPS rats (Fig. 1B,D). Furthermore, LPS-induced tachycardia was blunted in SAD + LPS in comparison to Control + LPS rats (Fig. 1B, P < 0.0001). Interestingly, SAD rats presented no fever, but a significant drop in Tb (hypothermia) in response to LPS (Fig. 1F,G, P = 0.0075). These data indicate that hemodynamic and thermoregulatory control during LPS-induced SI depends on the sino-aortic afferents integrity. Successfulness of SAD surgery was confirmed using pharmacological activation of the baroreflex with phenylephrine (Fig. 1C,E, P < 0.0001). The bradycardic baroreflex gain was calculated as ΔHR/ΔMAP (in beats per minute per millimeter of mercury).

### Variability in HR and systolic arterial pressure during LPS-induced SI

**Figure 2:**
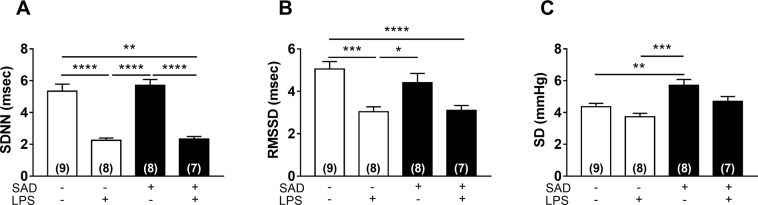
Variability in heart rate (HR) and in systolic arterial pressure (SAP) in the time-domain during LPS-induced SI. Standard deviation (SDNN, panel A) and root mean square of the successive differences (RMSSD, panel B) from PI series. Standard deviation (SD) from series (panel C). Control + Sal, Control + LPS, SAD + Sal, and SAD + LPS groups were evaluated 180 min after LPS administration. Results are presented as individual values and mean ± SEM. *p < 0.05, **p < 0.01, ***p < 0,001, ****p < 0.0001 using the one-way ANOVA with Tukey’s post hoc test. Control + Sal (*n* = 9), Control + LPS (*n* = 8), SAD + Sal (*n* = 9), and SAD + LPS (*n* = 7).

Variability of pulse interval in the time domain, determined by the standard deviation of normal to normal pulse interval (SDNN) and root mean square of successive differences (RMSSD), was significantly reduced in Control + LPS (P < 0.0001 and P = 0.0008) and SAD + LPS (P < 0.0001 and P = 0.0017) in comparison with Control + Sal (Fig. 2A,B). Variability of systolic arterial pressure in the time domain [evaluated by standard deviation (SD) of systolic arterial pressure] was significantly increased in SAD + Sal (P = 0.007), but not in SAD + LPS (P > 0.8305) in comparison with Control + Sal animals (Fig. 2C).

**Figure 3:**
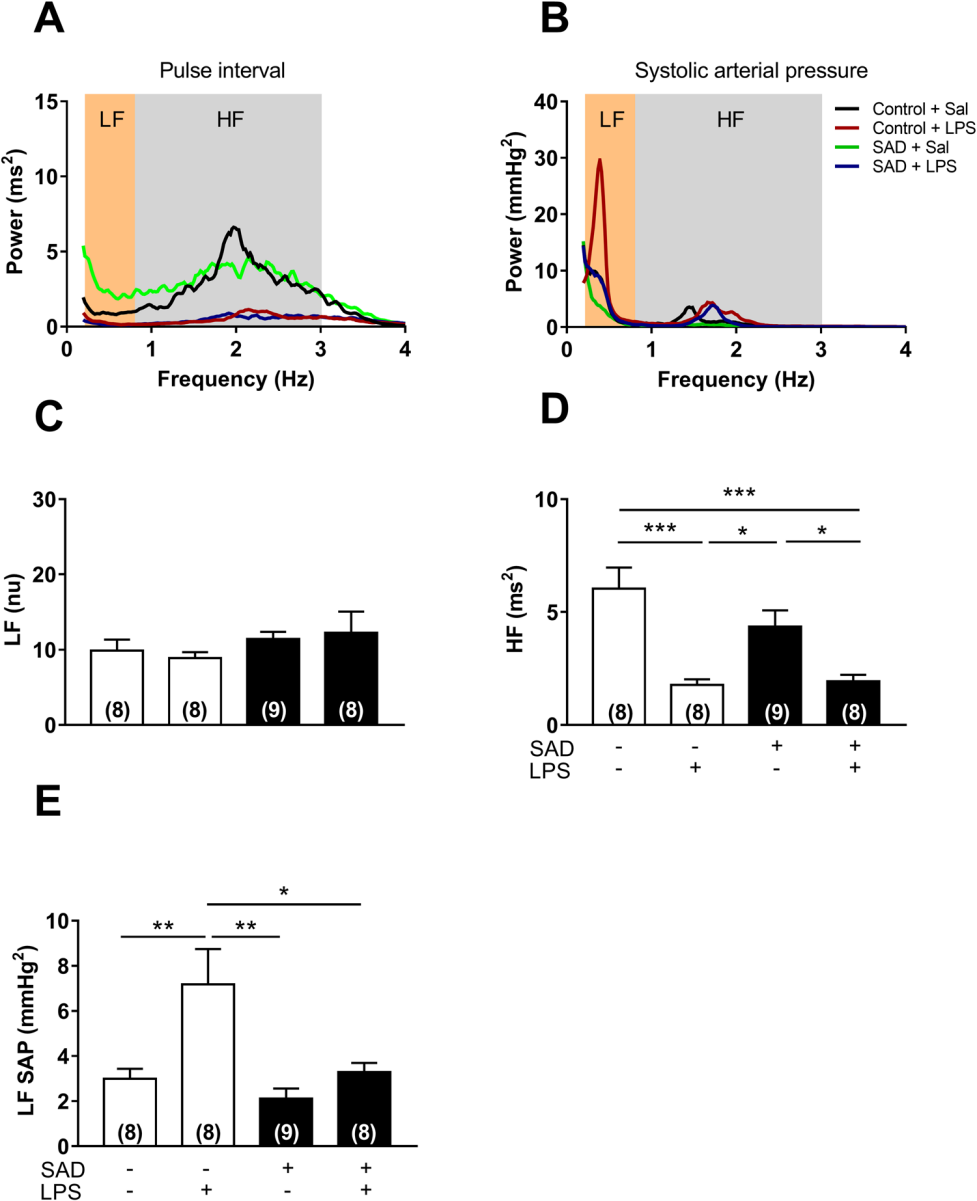
Power spectral analyses of pulse interval (PI) and systolic arterial pressure (SAP) from Control + Sal, Control + LPS, SAD + Sal, and SAD + LPS groups. Representative tracings from each experimental group (panel A and B). Magnitude of low frequency (LF, panel C) and high frequency (HF, panel D) components of PI. Magnitude of HF component of LF component of PI (panel E). Results are presented as mean ± SEM. *p < 0.05, **p < 0.01, ***p < 0,001, and ****p < 0.0001 using the one-way ANOVA with Tukey’s post hoc test. Control + Sal (*n* = 8), Control + LPS (*n* = 8), SAD + Sal (*n* = 9), and SAD + LPS (*n* = 8).

Spectral analysis in the frequency domain of the pulse interval showed that LF power was not significantly altered between Control + Sal, Control + LPS, SAD + Sal and SAD + LPS groups (Fig. 3A,C, P = 0.4904). Otherwise, HF power was significantly reduced in Control + LPS (P < 0.0003) and in SAD + LPS rats (P < 0.0005) in relation to Control + Sal group (Fig. 3B,D). These results indicate that LPS administration decreases cardiac vagal modulation in Control and SAD rats. Considering the systolic arterial pressure spectral analysis, a significant increase in the LF component was observed in the Control + LPS (P = 0.0096), but not in SAD + LPS (P = 0.9951) in relation to Control + Sal group. It was also observed a significant increase in the LF component in the Control + LPS in comparison with SAD + LPS group (P = 0.0172) indicating that LPS-induced increase in the sympathetic vasomotor modulation depends on the baroreceptors afferents integrity (Fig. 3B,E).

**Figure 4:**
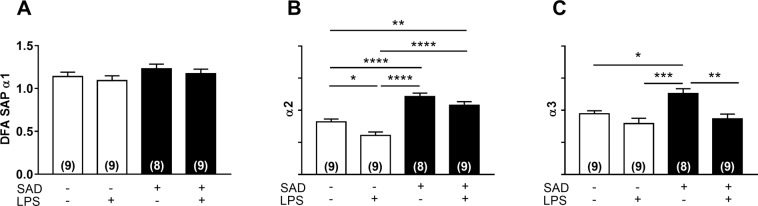
Detrended fluctuation analysis (DFA) from Control + Sal, Control + LPS, SAD + Sal, and SAD + LPS groups that where evaluated 180 min after LPS administration. Average values of α1 (panel A), α2 (panel B) and α3 (panel C) are shown. Results are presented mean ± SEM. *p < 0.05, **p < 0.01, ***p < 0,001, ****p < 0.0001 using the one-way ANOVA with Tukey’s post hoc test. Control + Sal (*n* = 9), Control + LPS (*n* = 9), SAD + Sal (*n* = 8), and SAD + LPS (*n* = 9).

**Figure 5:**
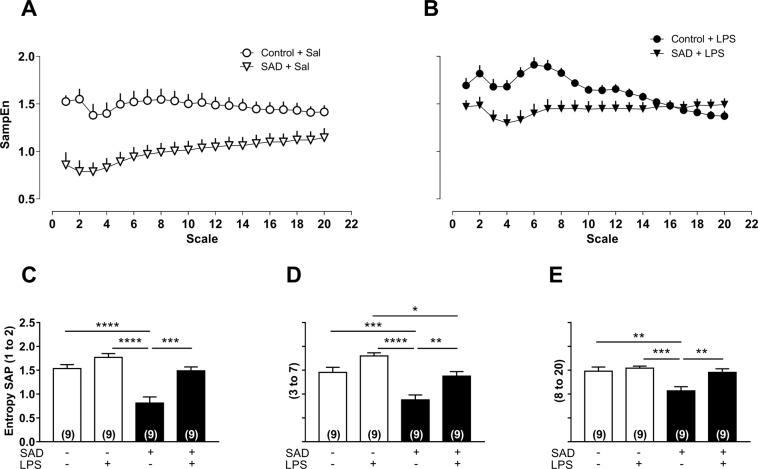
Multiscale entropy (MSE) from Control + Sal, Control + LPS, SAD + Sal and, SAD + LPS groups that where evaluated 180 min after LPS administration. Mean MSE profiles obtained from Control + Sal, SAD + Sal (panel A), Control + LPS, and SAD + LPS (panel B). Average values of entropy calculated for scales 1 and 2, 3 to 7 and 8 to 20 are shown (panel C). Results are presented as mean ± SEM. *p < 0.05, **p < 0.01, ***p < 0,001, ****p < 0.0001 compared using the one-way ANOVA with Tukey’s post hoc test. Control + Sal (*n* = 9), Control + LPS (*n* = 9), SAD + Sal (*n* = 9), and SAD + LPS (*n* = 9).

The detrended fluctuation analysis α_2 scaling exponent was lower in Control + LPS than in the Control + Sal group (P = 0.0355, Fig. 4B), whereas the same scaling exponent was significantly increased in SAD + Sal (P < 0.0001), in SAD + LPS (P = 0.0040) in relation to Control + Sal, and in SAD + LPS in relation to Control + LPS rats (P < 0.0001, Fig. 4B). The detrended fluctuation analysis α_3 scaling exponent was significantly higher in SAD + Sal (P = 0.0218) than in Control + Sal rats. Interestingly, the same scaling exponent was significantly reduced in SAD + LPS in comparison with SAD + Sal animals (P = 0.003, Fig. 4C). The multiscale entropy curves from all the evaluated groups are shown in Fig. 5A,B. The multiscale entropy for the SAD + Sal group was significantly reduced on small time scales in relation to Control + Sal (P < 0.0001; P = 0.0008 and P = 0.003; Fig. 5A,C,D,E). On the other hand, in the Control + LPS, the multiscale entropy was significantly increased in comparison with SAD + LPS group (P = 0.0173, Fig. 5B,D). Altogether these findings suggest that SI in the absence of a working baroreflex affects the nonlinear dynamics of cardiovascular function.

### Vascular reactivity during LPS-induced SI

**Figure 6:**
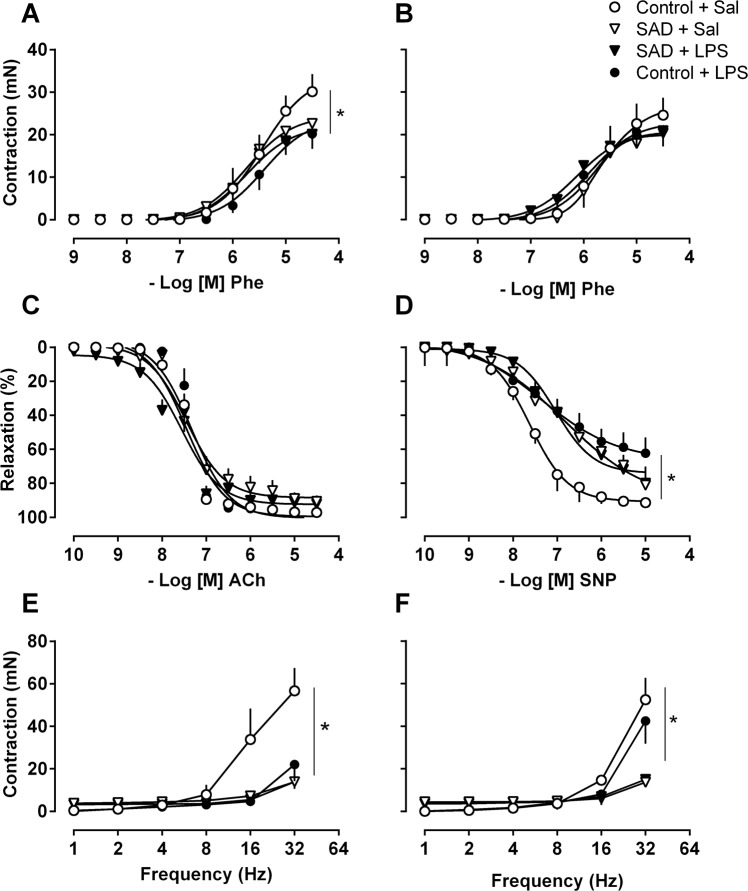
Cumulative concentration-response curves to the α-1 adrenergic agonist [phenylephrine (Phe) panel A], Phe in presence of L-NAME, non-selective inhibitor of nitric oxide synthase, (10^−4^ M, panel B), Acetylcholine (ACh), endothelium-dependent vasodilator (panel C), sodium nitroprusside (SNP) endothelium-independent vasodilator (panel D). Electrical-field stimulation (EFS, panel E) from Control + Sal, Control + LPS, SAD + Sal and, SAD + LPS groups. EFS in presence of L-NAME (10^−4^ M, panel F) in resistance mesenteric arteries. Results are presented as mean ± SEM. *p < 0.05 using the one-way ANOVA Tukey’s post hoc test. Control + Sal (*n* = 4–5), Co*n*trol + LPS (*n* = 5), SAD + Sal (*n* = 4), and SAD + LPS (*n* = 5).

Considering that LPS induced a significant drop in MAP associated with autonomic dysfunction and marked changes in cardiovascular complexity, we investigated whether SI leads to vascular damage in resistance blood vessels and if this eventual vascular dysfunction is exacerbated in rats submitted to SAD. Maximum contractile responses induced by phenylephrine were reduced in mesenteric arteries from Control + LPS and SAD + LPS groups (P < 0.05, Fig. 6A). Vascular hyporesponsiveness to vasoconstrcitors was reverted by the incubation with a non-selective inhibitor of nitric oxide synthase, L-NAME (P > 0.05, Fig. 6B). These data indicate that both SAD and LPS administration *per se* leads to vascular dysfunction, but without additive effects.

Furthermore, endothelium-dependent vascular relaxation induced by cumulative concentrations of acetylcholine was similar among the groups (Fig. 6C). In contrast, endothelium-independent vasodilation to sodium nitroprusside (SNP) was reduced in mesenteric arteries from Control + LPS, SAD + Sal, SAD + LPS groups in comparison with the Control + Sal group (P < 0.05, Fig. 6D). In addition, maximum contractile responses induced by electrical-field stimulation were significantly decreased in arteries from Control + LPS, SAD + Sal and SAD + LPS rats (P < 0.05, Fig. 6E). L-NAME reversed decreased electrical-field stimulation-induced contractions only in arteries from the Control + LPS group (P < 0.05, Fig. 6F).

### Cytokine levels in plasma and spleen during LPS-induced SI

Considering our previous study that documented hemodynamic and inflammatory changes 180 min following LPS administration^19^, cytokine levels were evaluated at this same period in plasma and spleen as an index of SI and the modulatory role of sino-aortic afferents on the splenic anti-inflammatory reflex, respectively.

#### Plasma

**Figure 7:**
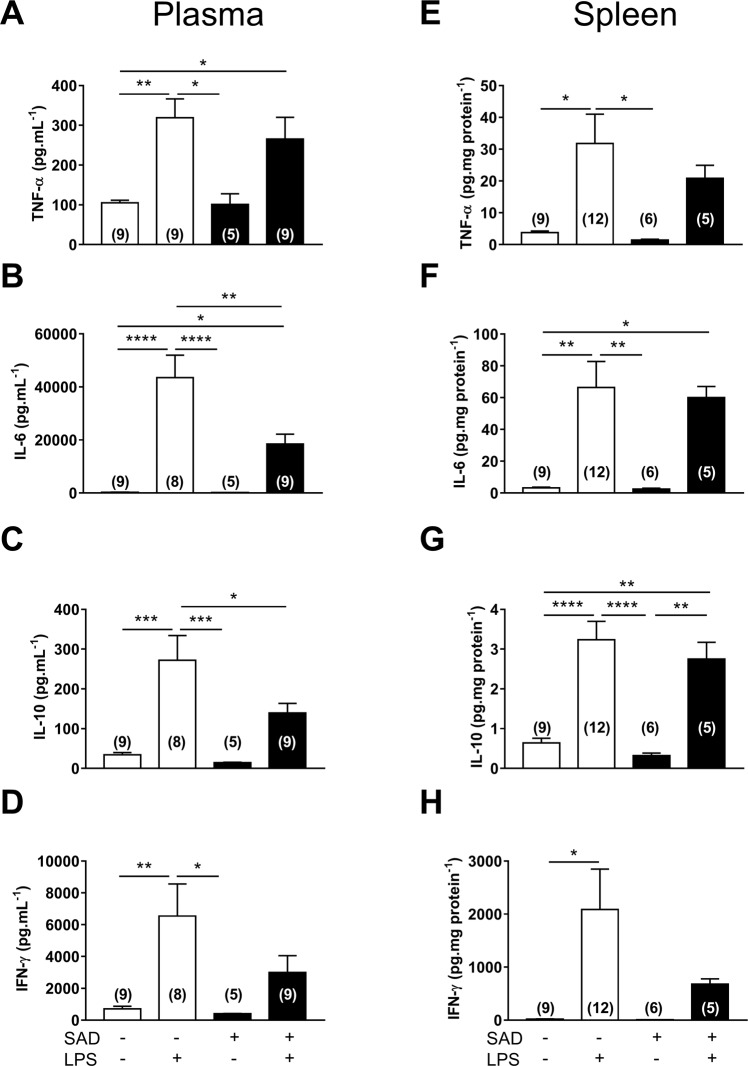
Plasma (panel A, B, C, and D) and splenic levels (**E**–**H**) of pro-inflammatory and anti-inflammatory cytokines from Control + Sal, Control + LPS, SAD + Sal and SAD + LPS groups. Results are presented as mean ± SEM. *p < 0.05, **p < 0.01, ***p < 0,001, ****p < 0.0001 using the one-way ANOVA with Tukey’s post hoc test. Control + Sal (*n* = 8–12), Control + LPS (*n* = 9–10), SAD + Sal (*n* = 5), and SAD + LPS (*n* = 7–13).

LPS increased the plasmatic levels of the pro-inflammatory cytokines TNF-α (P = 0.0036), IL-6 (P < 0.0001), and IFN-γ (P = 0.0074) and the anti-inflammatory cytokine IL-10 (P = 0.0002) in Control + LPS in comparison with Control + Sal rats. Interestingly, SAD decreased LPS-induced IL-6 (P = 0.0043) and IL-10 plasma surges (P = 0.0458, Fig. 7A–D). These results indicate that baroreflex positively modulates LPS-induced peripheral cytokine surges.

#### Spleen

Spleen is considered the efferent component of the “splenic anti-inflammatory reflex”^21^. In the spleen, we observed a significant increase in TNF-α (P = 0.0199), IL-6 (P = 0.0023), IL- 10 (P < 0.0001), and IFN-γ (P = 0.0399) levels in Control + LPS in comparison with Control + Sal rats. In addition, we observed reduced surges of TNF-α (P = 0.4464) or IFN-γ levels (P = 0.8898) in SAD + LPS group in relation to Control + LPS animals (Fig. 7E–H). These findings indicate that SAD reduces inflammatory signaling in spleen during SI.

### Corticosterone, NOx, and norepinephrine during LPS-induced SI

**Figure 8:**
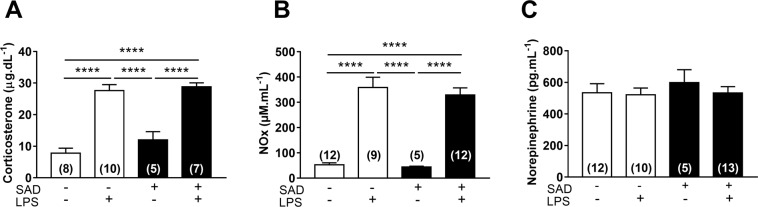
Plasma levels of corticosterone (panel A), NOx (nitrate, panel B), and norepinephrine (panel C) from Control + Sal, Control + LPS, SAD + Sal and SAD + LPS groups. Results are presented as mean values ± SEM. ****p < 0.0001 using the one-way ANOVA with Tukey’s post hoc test. Control + Sal (*n* = 9), Co*n*trol + LPS (*n* = 8–9), SAD + Sal (*n* = 5–6), a*n*d SAD + LPS (*n* = 5–9).

Plasma corticosterone levels were increased in Control + LPS (P < 0.0001) and in SAD + LPS (P < 0.0001) in comparison with Control + Sal group. There was no significant difference between Control + LPS and SAD + LPS groups (P = 0.9606, Fig. 8A). Interestingly, the observed LPS-induced drop in MAP was accompanied by increased plasma nitrate concentration, and these changes were independent of baroreceptor afferents integrity (P < 0.0001, Fig. 8B). Furthermore, plasma norepinephrine levels were similar in all the evaluated groups (Fig. 8C, P = 0.8499).

## Discussion

The present study is the first to report that baroreceptors afferents are key to modulate not only LPS-induced SI, but also its consequent cardiovascular changes and to provide evidence that these phenomena may not be directly dependent of each other. Supporting this notion, we observed that the LPS-induced drop in MAP was higher and earlier in rats previously submitted to SAD than in control rats. We also show that cardiac parasympathetic tone was reduced in rats that received LPS, while LPS-induced increase in sympathetic vasomotor tone was dependent on sino-aortic afferents integrity, even in the presence of vascular dysfunction. Of particular importance, we documented reduced surges of plasma interleukin (IL)-6 and IL-10 and splenic TNF-α and interferon-γ in SAD rats, indicating that SAD not only affected LPS-induced cardiovascular collapse but also reduced peripheral cytokines surges. Moreover, the reduced LPS-induced splenic cytokines surges seems to be at least in part mediated by the splenic anti-inflammatory reflex^21^, since reduced levels of TNF-α in the spleen was observed after SAD (Fig. 7).

Baroreceptor afferents integrity is important in the moment-to-moment control of cardiovascular function in physiological conditions^22^. During SI, LPS-induced drop in MAP was significantly enhanced after SAD (Fig. 1). In contrast, LPS-induced tachycardia was blunted in SAD rats. Tachycardia during SI is a compensatory mechanism regulating hypotension^18^ which is associated with vascular dysfunction^23^. Blunted tachycardia with greater drop in MAP in SAD rats may be related, at least in part, to a baroreflex-dependent cardiovascular regulation during SI^11,24,25^. Our results are consistent with the notion that the baroreceptors afferents integrity are involved, at least in part, in the LPS-induced drop in MAP (Fig. 1) caused by a reduction in vascular resistance (Fig. 6) even in the presence of increase of sympathetic vasomotor tone (Fig. 3).

LPS-induced SI reduced HR variability in the time-domain, which was not affected by SAD (Fig. 2). These findings are consistent with the notion that during SI, an imbalance in the autonomic cardiac control takes place independently of the sino-aortic afferents integrity. On the other hand, systolic arterial pressure variability was only increased in SAD group that received saline, suggesting that the hallmark of SAD experimental model, [higher variability of blood pressure^14^] is critically affected during SI.

In the search for mechanisms underlying hemodynamic control during SI, we used spectral analysis of cardiovascular function in the frequency domain, which showed that high frequency component of pulse interval was significantly reduced during SI (Fig. 3A,D). These observations are in line with the concept that LPS-induced tachycardia is triggered by mechanisms resulting in a decrease of vagal modulation to the heart^19^, which have been observed during the initial phase of sepsis-associated SI as a compensatory adjustment to avoid circulatory shock^26^. Regarding the variance of systolic arterial pressure in the frequency domain, the low frequency component was significantly increased in Control + LPS rats than in Control + Sal and SAD + LPS (Fig. 3), indicating that LPS-induced increases in sympathetic vasomotor tone depends on sino-aortic afferents integrity even in the presence of vascular dysfunction (Fig. 6). This eventual sustained sympatho-excitation during SI indicates that sympathetic drive to resistance vessels is not able to revert hypotension in this experimental model.

In addition to linear methods (time and frequency-domain analyses), non‐linear approaches were also used in the present study. Methods for analysis of nonlinear dynamics has been utilized to increase the interpretation of the complexity of the cardiovascular function^27,28^. We found that detrended fluctuation analysis α_2 scaling exponent of systolic arterial pressure was reduced in Control + LPS rats and was greater in SAD + Sal and in SAD + LPS than in Control + Sal rats (Fig. 4). These findings are consistent with the notion that the control of blood pressure is highly complex and that during LPS-induced SI the oscillations in systolic arterial pressure tend to erratic or random patterns (α_2 < 1). In contrast, when LPS is administrated to SAD animals, systolic arterial oscillations tend to be smoother (α_2 > 1). Reconciling the data obtained from multiscale entropy curves, we observed that systolic arterial pressure entropy of the Control + LPS was significantly different from those for SAD + LPS group (Fig. 5), suggesting the that the baroreflex plays an important role in the complex response to challenges, such as SI, imposed to the cardiovascular system^27^. Although a direct interpretation of the functional meaning of these results is not an easy task, the analyses of systolic arterial pressure complexity has been used to predict cardiovascular outcomes and has been associated with high mortality risks^29^. As far as we know, this is the first study that analyzed these cardiovascular complexity patterns by nonlinear approaches during SI in SAD rats. Whether or not SAD-induced changes in cardiovascular complexity may worse hemodynamic collapse during SI is an interesting matter deserving further investigation. Clinical trials are also needed to examine if these changes in cardiovascular complexity may predict outcomes in humans with sepsis.

Considering previous studies^18,19,30^ and our own data (Fig. 1) documenting cardiovascular collapse during SI, we further evaluated the vascular function after LPS administration in Control and SAD animals in mesenteric resistance arteries. After LPS administration, a significant reduction in contraction induced by phenylephrine and by electrical-field stimulation in mesenteric arteries from LPS-treated Control and SAD groups occurs (Fig. 6). Vascular hyporesponsiveness was reverted by the nitric oxide synthase inhibitor L-NAME, indicating that LPS-induced SI reduces contraction of resistance arteries by mechanisms that involve nitric oxide synthase activation whereas may be independent of baroreceptors afferents integrity. It is important to point out that sympathetic vasomotor tone was increased in Control + LPS conscious rats and that vascular responsiveness to phenylephrine was decreased in mesenteric resistance arteries of these animals in comparison with Control + Sal suggesting that in SI changes in the sympathetic nerves modulation to alpha1‐adrenergic receptors in mesenteric resistance arteries take place.

Additionally, the endothelium-dependent relaxation response induced by cumulative concentrations of acetylcholine was similar between the all Control and SAD groups (Fig. 6), indicating that neither SAD itself nor LPS administration caused endothelium dysfunction in our experimental condition. Conversely, endothelium-independent relaxation response was reduced in mesenteric arteries from all the groups that received LPS, probably due to changes in the sensitivity to guanylate cyclase/cGMP pathway^31^. The hypothesis that the guanylate cyclase/cGMP pathway is affected during SI is possible given that NO-induced activation of guanylate cyclase enzyme in vascular smooth muscle cells leads to recruitment of intracellular signaling cascades that reduce intracellular Ca^2+^ levels, open K^+^ channels and cause relaxation^32^.

Interestingly, rats submitted to SAD presented no fever, but a significant fall in Tb (hypothermia) in SI (Fig. 1). The mechanisms involved in LPS-induced hypothermia are not completely known, even though a previous study has documented the role of the ventral border of the dorsomedial nucleus in LPS-induced hypothermia^33^. SI-associated hypothermia has considerable clinical implications^34^. One may consider LPS-induced hypothermia as a failure in neural control of Tb. Alternatively, recent studies have suggested that hypothermia is precisely controlled by specific mechanisms mediated by the central nervous system^35^. Based on these data, we suggest that baroreceptor afferents integrity affect thermoregulatory control during SI by impairing a key part of the afferent signals to the brain. We further speculate that among the important multimodal functions of arterial baroreceptors^22^ febrigenic signaling in the periphery affects brain circuitry at least in part by interacting with peripheral baroreceptors afferents. The reduced LPS-induced plasma surges of IL-6 in SAD rats (Fig. 7) may be at least one of the contributing factors in thermogenesis during SI, since this cytokine is known to induce increases in Tb acting as an important endogenous pyrogen^36^. These baroreceptor afferents effects on thermoregulation must take place through thermoeffectors modulation, but it remains unknown if this modulation is via sympathetic innervation of the brown adipose tissue (affecting non-shivering thermogenesis) or sympathetic innervation of the tail artery (affecting heat loss index).

The LPS-induced plasma surges of IL-6 (a pro-inflammatory cytokine) and IL-10 (an anti-inflammatory cytokine) in SAD rats were significantly reduced after SAD (Fig. 7). These findings indicate that baroreflex does modulate the LPS-induced peripheral cytokine surges and adds new information to a previous study that documented that electrical baroreflex stimulation inhibits LPS-induced pro-inflammatory cytokines surges not in the periphery but in the brain^20^. A putative mechanism by which sino-aortic afferents may play a modulatory effect in the LPS-induced IL-6 and IL-10 plasma surges may be related to macrophages polarization, a highly heterogeneous cell population, during SI. Activated macrophages M1 exhibit high levels of pro-inflammatory cytokines, while activated M2 macrophages exhibit high levels of anti-inflammatory cytokines^37^.

In addition to circulating macrophages aforementioned, the main efferent target organ for the splenic anti-inflammatory reflex is the splenic macrophages located in the white pulp^2^. We showed here that spleen tissue homogenates collected from rats that received LPS exhibit a significant increase of pro-inflammatory cytokines. Surprisingly, there were no significant increases in TNF-α [an essential early mediator of inflammation^2,38^] or IFN-γ [a later inflammatory marker^39^] levels in rats submitted to SAD during LPS-induced SI (Fig. 7). Combining a previous study showing that stimulation of the efferent fibers impinges upon the spleen leads to a significant anti-inflammatory effects in this organ during LPS-induced SI^40^ and our own data in which SAD rats showed decreased LPS-induced surges of pro-inflammatory cytokines in the spleen, we suggest that the efferent arm of the splenic anti-inflammatory reflex is modulated by baroreceptors afferents.

Considering that baroreflex stimulation downregulates pro-inflammatory cytokines in hypothalamus, but not in plasma, heart and spleen^20^ we hypothesized that SAD worsens cytokines surges in plasma. Contrary to our expectations, after SAD, LPS-induced surges of cytokines were blunted in plasma and spleen (Fig. 7) suggesting that the baroreceptor afferents integrity/stimulation may differentially affect peripheral and central pro-inflammatory cytokines surges in this critical condition that resembles some features of sepsis, a considerable healthcare burden.

A plethora of studies have provided strong evidence demonstrating autonomic regulation of immune function^2,41^. For instance an inhibitory action of the sympathetic nervous system and its main neurotransmitter, norepinephrine on SI has been documented^2,41^. In the present study, we show that the known LPS-induced enhancement of sympathetic vasomotor tone may be attributable, at least in part, to sino-aortic afferents integrity (Fig. 3). Whether the LPS-induced sustained increase in the activity of the splenic nerves^2^ is affected by sino-aortic afferents is a possibility that requires additional investigation. Given the vagal withdrawal during SI one may speculate that SAD counteracts this autonomic adjustment and limits inflammation. In addition, if pro-inflammatory cytokines surges in SI^2^ are regulated by baroreceptor afferents or if this exacerbate release of immune mediators represents a failure of the adaptive mechanisms are interesting matters to be explored.

In SI, a significant increase in plasma corticosterone (a hormone with anti-inflammatory action^42^) levels occurs. This LPS-induced increased corticosterone levels were not affected in SAD rats (Fig. 8A). These data support the notion that: (1) during LPS-induced SI an increase in the hypothalamic-pituitary-adrenal axis activity occurs, (2) this activation is independent of the baroreceptor afferents integrity and, (3) the reduction in the cytokines surges in SAD was not due to the down regulation of the hypothalamic-pituitary-adrenal axis.

To provide insights into the mechanisms involved in hypotension during SI, we also assessed plasma NO (a potent vasodilator) and norepinephrine (a vasopressor neurotransmitter) levels (Fig. 8). Taking into consideration that during sepsis, NO pathway system is markedly stimulated leading to decreased vascular responsiveness to constrictor stimuli^23^, our findings further support the notion that indeed LPS-induced SI is accompanied by a significant increase in plasma NO production, that does not depend on baroreceptors integrity. Moreover, these findings indicate that greater hypotension in SAD rats is not associated with higher NO production systemically, but rather to the autonomic imbalance *per se* (Fig. 8B). Similarly, the observed effects of SAD on the LPS-induced hemodynamic dysfunction seems to be independent of systemic noradrenaline levels, since this catecholamine levels were similar among groups (Fig. 8C). However, these data do not rule out that local noradrenaline release from sympathetic nerve terminals during SI may be different depending on the vascular bed.

Our study had several limitations: (i) Even though we observed that SAD affects LPS-induced cardiovascular collapse, thermoregulation and inflammatory signaling, we can make no conclusions about the causal link between these important regulatory functions, reflecting the complexity of this experimental model in which a myriad of events lead to multiple organ failure and eventually death depending on the doses of LPS. We suggest that the baroreflex-dependent mechanisms mediating inflammatory status are not associated with cardiovascular collapse, given that SAD reduced cytokines surges (both in plasma and spleen) and exacerbate hypotension. (ii) In the present study we used a single dose of LPS based in on our experience to induce septic-like non-lethal cardiovascular collapse. It is possible that baroreceptor afferents display different roles during SI due to lower or higher (lethal) doses of LPS. For this reason, a survival experiment was not done. (iii) We cannot draw conclusions about the cell type responsible for splenic TNF-α and interferon-γ reduction in SAD rats. We also used the plasma cytokines surges analysis and we can suggest that reduced splenic cytokines levels was local. (iv) We expected a significant decrease in vascular reactivity in SAD rats treated with LPS. However, SAD *per se* leads to changes in vascular function suggesting that SAD and LPS-induced SI are both complex models in the study of vascular function. It is worth mentioning that in the vascular reactivity experiments there were no circulating inflammatory cells as in conscious animals. All in all, it is well known that some days after SAD, different compensatory mechanisms take place. (v) We used the well accepted model of endotoxemia and our results cannot be directly extrapolated for polymicrobial sepsis.

In conclusion, the present data are consistent with the notion that the role of baroreflex afferents on LPS-induced SI goes beyond the lessening hypotension and tachycardia despite severe vascular dysfunction and affecting inflammatory status. The present findings shed light on the mechanisms underlying the contribution of cardiovascular afferents in the regulation of the inflammatory surges in plasma and spleen during SI. This study provides evidence that baroreceptor afferents are involved in LPS-induced SI and cardiovascular collapse.

## Materials and Methods

All experiments were executed according to directions for National Council for Animal Experimentation Control in Brazil (CONCEA). The experimental procedures were also approved by The Ethics Committee on Animal Research of the Dental School of Ribeirão Preto - University of São Paulo, Ribeirão Preto, Brazil (#2017.1.585.58.9).

### Animals

We used 58 adult male Wistar rats (300–350 g) that were acquired from the Animal Care Facility of the University of São Paulo at Ribeirão Preto. They were kept in the animal facility of the Dental School of Ribeirão Preto, University of São Paulo under a 12-h light/dark cycle (lights on at 6 am) at 23–24 °C, and unrestricted access to standard chow and tap water.

### Surgical procedures

The most well accepted model of baroreceptor afferents removal, sino-aortic denervation (SAD)^25,43,44^, was performed aseptically using a standard technique^45^. Rats were anesthetized with ketamine (100 mg.kg^−1^) and xylazine (10 mg.kg^−1^) and fixed in the supine after the absence of the withdrawal reflex to tail and paw pinch. Additional doses of anesthetic were administrated if necessary. A ventral midcervical incision was performed and fibers from the aortic depressor nerve traveling with the superior laryngeal nerve and superior cervical ganglion were transected. The carotid baroreceptors were denervated by removal of surrounding tissues from the carotid sinus.

On the fourth day after SAD rats were anaesthetized with ketamine and xylazine and a polyethylene catheter (PE-10 connected to PE-50 tubing; Clay Adams, Parsippany, NJ, USA, Intramedic, Becton Dickinson, Sparks, MD, EUA), was placed into the abdominal aorta by means of femoral artery for direct hemodynamic recordings. Femoral vein was also catheterized for drug administration. Both catheters were tunneled subcutaneously and exteriorized through the skin in the nape of the neck, and the surgical wounds were sutured. Rats recovered individually in the recording room. On the following day the arterial catheter was connected to a pressure transducer (MLT0380; ADInstruments), and in turn, to an amplifier (Bridge Amp, ML221; ADInstruments). Pulsatile arterial pressure (PAP) and heart rate (HR) were recorded using the Chart Pro software (ADInstruments). Rats from different groups were recorded simultaneously placed in side-by-side cages. Beat-by-beat series of systolic arterial pressure and pulse interval were obtained from the raw PAP recordings and systolic arterial pressure or pulse interval variability was evaluated using the software CardioSeries^46^ and JBioS^47^.

In the time domain, standard deviation (SDNN) and root mean square of the successive differences (RMSSD) were calculated from pulse interval series. Standard deviation (SD) was also obtained from systolic arterial pressure series. In the frequency domain, the power spectra of pulse interval and systolic arterial pressure were estimated by the modified periodogram and Welch protocol^48^. Briefly, all series were interpolated at 10 Hz (cubic spline) and divided into segments of 512 points (51.2 seconds). Segments containing artifacts or transients were excluded. Next, each selected segment was multiplied by a Hanning window and the periodogram was estimated. The pulse interval spectra were integrated into low- (LF, 0.2–0.75 Hz) and high-frequency (HF, 0.75–3 Hz) bands, while the systolic arterial pressure spectra were integrated at LF band only. The power at LF band was assessed in normalized units (nu), represented by LF/(LF + HF), whereas the power at HF band was evaluated in absolute units. According to previous studies^28,49^, this representation provides the best correlation of spectral indices to the sympathetic and parasympathetic modulation of the heart rate, respectively.

Nonlinear properties of pulse interval and systolic arterial pressure series were assessed by multiscale entropy and detrended fluctuation analysis^50,51^. Multiscale entropy quantifies the degree of irregularity (unpredictability) of time series over increasing time scales and can be considered a measure of physiological complexity. Healthy systems represent the most complex physiological status, whereas aging and diseases denote some disruption in the integrative regulatory mechanisms, decreasing the capability of the organism to adapt to changing demands^52^. Multiscale entropy parameters were set to m = 2 (embedding dimension), r = 15% of time series SD (tolerance factor) and τ = 1…20 (time scales). On the other hand, detrended fluctuation analysis quantifies the power law scaling of time series, which is related to its fractal temporal structure^53^. In the present study, α_1 comprises windows from 5 to 15 points and α_2 comprises windows from 30 to 100 and α_3 comprises windows from 100 to N⁄10 points, where N is the time series length.

In the same surgical procedure for arterial and venous catheterization, a median laparotomy was done and an intraperitoneal temperature data-logger capsule (SubCue, Calgary, AB, Canada) was inserted to deep body (Tb) temperature recordings in rats. Afterward, surgical wounds were sutured aseptically.

### Vascular reactivity studies

The method described by Mulvany and Halpern^54^ was used. Animals were euthanized and segments of third-branch mesenteric arteries, measuring about 2 mm in length, were mounted in a small vessel myograph (Danish Myo Tech, Model 620 M, A/S, Århus, Denmark). Arteries were maintained in a Krebs Henseleit solution [(in mM) NaCl 130, KCl 4.7, KH_2_PO_4_ 1.18, MgSO_4_ 1.17, NaHCO_3_ 14.9, Glucose 5.5, EDTA 0.03, CaCl_2_ 1.6], at a constant temperature of 37 °C, pH 7.4, and gassed with a mixture of 95% O_2_ and 5% CO_2_.

Mesenteric resistance arteries were set to reach a tension of 13.3 kPa (kilopascal) and remained at rest for 30 min for stabilization. The arteries were stimulated with Krebs solution containing a high concentration of potassium [K^+^, (120 mM)] to evaluate the contractile capacity of the segments. After washing and return to the basal tension, arteries were contracted with phenylephrine (10^−6^ M) and then stimulated with acetylcholine (10^–5^ M) to determine the presence of a functional endothelium. Arteries exhibiting a vasodilator response to acetylcholine greater than 80% were considered endothelium-intact vessels^55,56^. After washing and another period of stabilization, concentration-response curves to phenylephrine (10^−10^ to 3 × 10^−5^) and electrical-field stimulation were performed in mesenteric resistance arteries to produce contractions, measured as increases in baseline tension. Electrical-field stimulation was applied to arteries placed between platinum pin electrodes and conducted at 20 V, 1-ms pulse width, and trains of stimuli lasting 10 s at varying frequencies (1 to 32 Hz).

Vasodilation responses were determined in mesenteric resistance arteries contracted with phenylephrine (10^−6^ to 3 × 10^−6^ M). After 15 min, concentration-response curves to acetylcholine (10^−10^ to 3 × 10^−5^ M) and sodium nitroprusside (10^−10^ to 10^−5^ M) were carried out. Concentration-response curves to phenylephrine, electrical-field stimulation and acetylcholine were also performed in the presence of L-NAME (10^−4^ M).

### Plasma measurements

At the end of the cardiovascular and Tb recordings, arterial blood was withdrawn in EDTA-coated tubes and centrifuged (20 min at 3.500 rpm, 4 °C), for plasma extraction, 180 min after saline or LPS administration. All plasma samples were kept at −80 °C until assays. Plasma samples were assayed for measurement of tumor necrosis factor (TNF)-α, interleukin (IL)-6, IL-10 and interferon (IFN)-γ using multiplex assay kits according to standard instructions (LXSARM - 05, R&D System, Minnesota, USA) with Luminex Magpix technology (Austin, TX, USA). Plasma samples were assayed for measurement of noradrenaline (Cloud-Clone, Texas – USA) levels, using enzyme-linked immunosorbent assay (ELISA) kits according to standard instructions. Spleens were homogenized in 0.5 mL of PBS, protease inhibitor cocktail (Cell Signaling, Massachusetts, USA) and then centrifuged at 13,000 rpm for 20 min at 4 °C. Tissue supernatant samples were used to measure TNF-α, IL-6, IL-10, and IFN-γ levels by a multiplex assay as in plasma samples. Data from splenic cytokines were normalized by protein concentrations by means of Bradford assay (#5000205, Bio-Rad Laboratories, USA). Plasma corticosterone extractions and radioimmunoassay were performed from 25 μL of plasma by adding 1 mL of ethanol according to Haack *et al*., (1979)^57^. Plasma NOx levels were assessed by using the chemiluminescence NO-Ozone technique. Nitrate concentrations were measured using 40 μL aliquots of the plasma samples inserted into a NO analyzer (Model 280, Sievers Instruments, Boulder, CO, USA).

### Experimental Protocols

1. To study the role of sino-aortic afferents integrity in LPS-induced changes in MAP, HR and Tb, rats were catheterized and had a datalogger implant and on the day after they received an iv injection of saline or LPS and were recorded up to 180 min after iv administration. To avoid the influence of variability of MAP of SAD rats in the results, the reported values of MAP and HR were obtained as the delta of beseline values (using a mean of 10 min of recording before the intravenous injection) and the minimum value for MAP and the maximum value for HR obtained from the last minute of every 10 min period throughout 180 min. This analysis was done in all the experimental groups.
2. To further characterize the role of sino-aortic afferents integrity in LPS-induced cardiovascular collapse, spectral analysis of pulse interval and systolic arterial pressure in the time and in the frequency domain, and analyze of the complexity of cardiovascular function were evaluated offline.
3. 180 min after LPS or saline administration, arterial plasma was withdrawn to assess corticosterone, NOx, and norepinephrine levels. Cytokines levels were also assessed in plasma and spleen.
4. 180 min after LPS or saline administration, rats were euthanized and vascular reactivity was evaluated in mesenteric resistance arteries.

### Statistical analysis

Data are expressed as mean ± S.E.M. (standard error of the mean) and significant differences were considered at P ≤ 0.05, but exact P values are described. Unpaired t test, one-, two-way ANOVA followed by the Tuckey’s multiple comparisons test were performed when necessary. For the statistical analysis we used GraphPad Prism (Version 7.03, La Jolla, CA, USA).
