# Author Correction: Baroreceptor denervation reduces inflammatory status but worsens cardiovascular collapse during systemic inflammation

**DOI:** 10.1038/s41598-020-69650-3

**Published:** 2020-07-28

**Authors:** Mateus R. Amorim, Júnia L. de Deus, Camila A. Pereira, Luiz E. V. da Silva, Gabriela S. Borges, Nathanne S. Ferreira, Marcelo E. Batalhão, José Antunes-Rodrigues, Evelin C. Carnio, Rita C. Tostes, Luiz G. S. Branco

**Affiliations:** 1 Dental School of Ribeirão Preto, University of São Paulo, São Paulo, 14040-904 Brazil; 2 Ribeirão Preto Medical School, University of São Paulo, Ribeirão Preto, São Paulo, 14049-900 Brazil; 3 Nursing School of Ribeirão Preto, University of São Paulo, Ribeirão Preto, São Paulo, 14040-902 Brazil

Correction to: *Scientific Reports* https://doi.org/10.1038/s41598-020-63949-x, published online 24 April 2020

This Article contains errors in the Methods section under subheading ‘Surgical procedures’.

“(PE-10 connected to PE-50 tubing; Clay Adams, Parsippany, NJ, USA, Intramedic, Becton Dickinson, Sparks, MD, EUA)”

should read:

“(PE-10 connected to PE-50 tubing; Clay Adams, Parsippany, NJ, USA, Intramedic, Becton Dickinson, Sparks, MD, USA)”

Additionally, in the Methods section, the subheading “Plasma Measurements” should read “Plasma and Spleen Measurements”.

